# Protamine and other proteins in sperm and seminal plasma as molecular markers of bull fertility

**DOI:** 10.14202/vetworld.2020.556-562

**Published:** 2020-03-25

**Authors:** Berlin Pandapotan Pardede, Muhammad Agil, Iman Supriatna

**Affiliations:** 1 Reproductive Biology Study Program, Faculty of Veterinary Medicine, IPB University, Bogor, Indonesia; 2 Department of Veterinary Clinic, Reproduction and Pathology, Division of Reproduction and Obstetric, Faculty of Veterinary Medicine, IPB University, Bogor, Indonesia

**Keywords:** fertility, molecular marker, protamine, seminal plasma, sperm proteins

## Abstract

Fertility is the most important aspect in the efforts to increase livestock populations. Protamine and various proteins in sperm and seminal plasma are the results of the molecular analysis which can be used as a marker of fertility. Each of the proteins plays an important role in the normal function of sperm, starting from the formation of sperm structure, motility, capacitation, cell protection, acrosome reactions, successful fertilization, egg activation, and embryonic development. Finally, these molecular components can be a marker of fertility and can help to diagnose the cases of infertility/subfertility in livestock in the field.

## Introduction

Fertility is closely related to the capacity of a bull to produce sperm that is able to fertilize oocytes and produce new individuals. Fertility is influenced by many factors, including management, age, nutrition, genetics, and disease. The decreased fertility in bulls will have an impact on the low level of conception/conception rate in cows and ultimately will reduce the productivity and economic problems in the livestock industry [1]. Various studies related to bull fertility have been carried out through various approaches, including genomics, transcriptomics, and proteomics. Genomics is a study related to the whole genome of an organism, transcriptomics is a study of the overall transcription product (RNA) of an organism, and proteomics is a study related to protein traits (level of expression, interaction, and modification after translation and others) on a large scale to gain clear insight and integrated [2]. Analysis with various approaches is used to ensure bull fertility in a molecular manner.

Protamine (PRM) is the main protein found in the nucleus of the sperm that binds DNA and is one of the molecular markers of fertility [3,4]. Analysis with various other molecular approaches has also found various molecular components in sperm and seminal plasma, which are significantly correlated in bull fertility [5-8]. Proteins or marker genes that are the result of molecular analysis are very useful in accurate bull selection in efforts to increase the livestock populations. Based on the case in the field, it is proven that some bulls are classified as low fertility even though in a standard evaluation of semen quality such as motility, viability, and abnormality shows normal levels [9].

Assembled studies of proteins or genes marking fertility in bulls need to be known, to see the potential of a bull in relation to fertility, and certainly can be used as a basis for bull selection in an effort to increase the livestock populations. Hence, this review will discuss PRM and other proteins in sperm and seminal plasma and their role in fertility.

### PRM and Other Sperm Proteins

**Figure-1:**
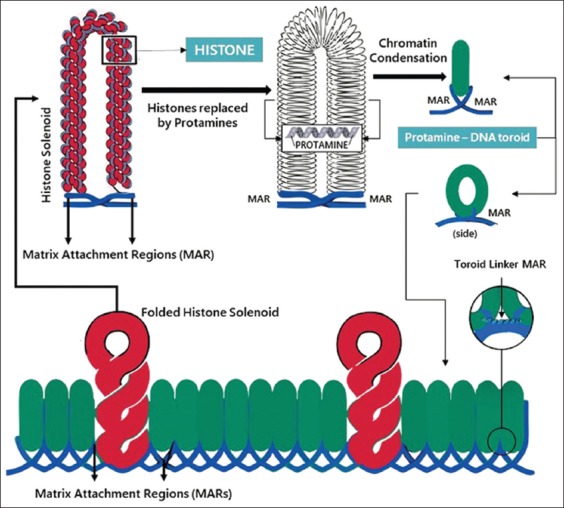
The sperm DNA organization is organized in the toroid form. Adapted from Ward [3].

Sperm protein has an important role related to fertility, such as morphological integrity and sperm function, including motility, capacitation, fertilization, oocyte activation, and embryonic development [10]. Sperm core structure is one of the important components in fertility. The sperm DNA material is packaged in the sperm core in a unique and complex system with special proteins that regulate the process of condensation and decompression through certain mechanisms [3]. Structurally, most of the sperm DNA is coiled into a toroid, a small portion is attached to histones and the rest is attached to the core matrix of sperm matrix attachment regions (Figure-1).

The densest DNA sperm organization is inside the toroid. During the process of maturation, most of the histone proteins associated with DNA are replaced by PRM. The main protein in the sperm nucleus that binds DNA is PRM [3]. During spermiogenesis, haploid spermatids undergo various complex physiological and morphological processes that eventually differentiate into sperm [11]. The process includes the sperm chromatin remodeling process which is characterized by the replacement of histones through PRM, and transition proteins (TNPs) 1 and 2. During the process of spermiogenesis, as much as 85% of histone sperm nuclei will be replaced by PRM [12]. Hong *et al*. [13] reported that the deactivation of TNPs would result in decreased fertility, increased DNA damage, and various other sperm morphological abnormalities. As reported by de Oliveira *et al*. [6], histones contained in the sperm nucleus have an important role in fertility in the bull.

**Figure-2:**
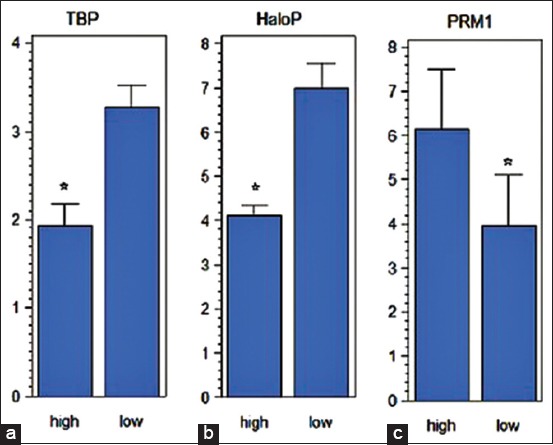
The level of DNA damage in bulls with high and low fertility with toluidine blue (a) and Halomax (b) staining; PRM1 expression in high and low fertility cattle (c) [4].

PRM plays an important role in the formation of chromatin, which is needed in normal sperm function. HM *et al*. [14] reported that abnormal expression of PRM caused a decrease in sperm count, motility, morphology, and increased sperm chromatin damage. Aoki *et al*. [15] also reported that there was a decrease in viability and increased DNA damage as a result of the abnormal expression of sperm PRM. PRM1 is the gene most widely expressed in cattle bulls and tends to decrease expression in bulls with low fertility [5]. Dogan *et al*. [4], through a proteomics approach, proved that PRM in sperm is correlated with bull fertility. PRM1 is highly expressed in bulls with high fertility and DNA damage in low sperm (Figure-2). These studies show that the number of histones, as well as the ratio of histones and PRM, will greatly determine the level of fertility of the bull.

In addition to PRM, there are other proteins in sperm, which play an important role in carrying out the normal function of the sperm and its relation to the process of fertilization, one of which is in the process of capacitation. Sperm capacitation plays an important role related to the ability of sperm to fertilize oocytes, which are various proteins involved in the capacitation. Peddinti *et al*. [16] reported that casein kinase II plays a role in the process of epidermal growth factor signaling at the time of sperm capacitation in Holstein bulls with different levels of fertility. Sperm phospholipase C zeta 1 (PLCZ1) is also reported as one of the fertility marker proteins in cattle. The protein is involved in intracellular Ca^2+^ oscillations that play a role during egg activation after fertilization [17]. The increased intracellular concentrations of Ca^2+^ caused by the production of inositol 1,4,5-triphosphate (IP3) will trigger an oocyte activity [18]. PLCZ1 plays an important role in the process of releasing Ca^2+^ and IP3 production [19]. The PLCZ1 encoding gene is located on the 5^th^ chromosome of bovine, and the genetic variation in the PLCZ1 promoter region is also related to semen quality [20]. Grant *et al*. [21] also reported that superoxide dismutase (SOD) in high fertility bulls is highly expressed. SOD is an antioxidant that has been proven to protect sperm from reactive oxygen species and can increase the sperm membrane integrity and motility in bulls [22].

Fertilin (PH-30) α and β are also reported as one of the proteins that are related to the fertility of bulls. The protein incorporated in the transmembrane protein family, the ADAMs gene (a disintegrin and metalloprotease domain), plays an active role in mediating the binding and fusion of the oocyte-sperm membrane. In cattle, the fertilin subunit α belongs to Adam 1 (17^th^ chromosome) and fertilin subunit β belongs to Adam 2 (27^th^ chromosome) [23,24]. Bahbahani *et al*. [25] reported that calmegin, which is located in the 17^th^ chromosome of bovine, is a fertility-related gene, in which the deactivation of this gene will have an impact on sterile bulls, and the disruption of the migration process into the oviduct and binding to the pellucid zone does not occur. Proteins or other genes are also reported as fertility-related proteins, such as zonadhesin [26] which play a role in binding to the pellucid zone, calcium, and c-AMP which play a role in sperm capacitation [23], and lactate dehydrogenase C which plays a role in controlling the production of ATP and its relation to sperm motility [27].

ENO1, MDH2, ubiquitin carboxyl-terminal hydrolase-12 (USP12), and RIBC have also been reported as fertility coding protein genes in crossed cattle [28]. ENO1 (enolase-1/alpha enolase) is an enzyme found in motile spermatozoa and has various functions [29]. The function of ENO1 is to regulate energy for sperm motility and helps to protect from oxidative stress [30]. Mitochondrial malate dehydrogenase-2 NAD (MDH2) is an enzyme that plays a role in the regulation of energy in sperm, and the abnormal expression of MDH2 will cause the disruption of internal energy distribution in spermatozoa and has an impact on sperm motility, the process of capacitation, the occurrence of hyperactivation, and decreased fertility [16]. USP12 plays an important role in stabilizing and enhancing the cellular function of androgen receptors, which are very important in regulating various sperm functions [31]. RIB 43 A-like with coiled-coils protein-1 (RIBC) is another protein that plays a role in fertility, specifically related to its function in motility [28]. The expression of RIBC protein in cross cattle will reduce the sperm’s functional ability to achieve fertilization [28]. Another protein in sperm has potential as a marker of fertility is outer dense fiber protein 2 (ODF2), which is present in the sperm tail structure and plays an active role in motility [32], and found not only in bulls but also in buffaloes [33]. Besides ODF2, sperm tails also have tubulin beta-2C chains that have decreased in asthenozoospermia [34]. Fu *et al*. [33] also reported several proteins found in buffalo sperm such as A-kinase anchor protein 4, which acted as the main structural component of sperm fibrous sheath; tubulin which plays a role in microtubules; and glutathione S-transferase Mu 3 and calmodulin which act as a mediated enzyme.

### Plasma Seminal Proteins

Bovine seminal plasma (BSP) is a protein that is secreted by the seminal vesicles and is closely related to the fertility of cattle bulls. These BSP encoding genes, include BSP1, BSP3, BSP4, BSP5, BSPH1, and BSPH2, are located in the 11^th^ chromosome in bull cattle [35,36]. On the sperm surface, BSP binds specifically with phosphatidylcholine, sphingomyelin, and plasmalogen, interacts with heparin and high-density lipoprotein, and capacitates factors in bulls [37]. Aslam *et al*. [28] reported that high BSP1 gene expression was found in cow sperm with low fertility. BSP1 plays a role in the regulation of sperm motility, sperm capacitation modulation, and acrosome reaction [38,39], whereas in buffalo, Brito *et al*. [40] also reported that BSP is the most common protein found in seminal plasma, including BSP5 and BSP3, and has the same role in metabolic processes as in bulls.

The high levels of BSP1 will cause an imbalance in the plasma membrane, so the sperm will be more sensitive during cryopreservation [41], and cause early capacitation of sperm, which will reduce or reduce the cryosurvivability and fertility of the bull [42,43]. Viana *et al*. [44] reported that high BSP5 concentrations are found in high fertility bulls, and this protein is very useful in the process of membrane stability and sperm capacitation, and embryonic development. Seminal vesicle also secretes bovine PLA2 protein, which is on the 16^th^ chromosome and plays a role in sperm acrosome reactions and capacitation process. Deactivation of the PLA2 gene will affect the disruption of sperm motility and reduce the fertility rates specifically [45]. Whey acidic protein and epididymal-specific lipocalin 5 which play a role in the process of sperm maturation and metabolism are also found to be abundant in buffalo seminal plasma [40]. Karunakaran *et al*. [46] reported that osteopontin which plays a role in binding sperm-oocytes and early embryonic development is also a marker of fertility in cattle. The osteopontin coding gene is located on the 6^th^ chromosome in bovine. In cattle, osteopontin binds to the sperm acrosome hood during ejaculation, and the bond is still present after sperm which comes in contact with oviduct secretion and experiences an acrosome reaction *in vitro* [47,48], whereas in buffalo, osteopontin is found to be quite high in seminal plasma and is associated with the process of capacitation, fertilization, and early embryonic development [40].

Spermadhesin 2 (spermadhesinZ13) which is a protein with a coding gene located on the 26^th^ chromosome in cattle is reported to have a negative impact on fertility, reduces sperm motility, and is expressed in many low fertility cattle [35,49]. Štiavnická *et al*. [50] found that a high ubiquitin protein in cow sperm is closely related to quality and low fertility in bulls. Another protein that is also found in seminal sperm plasma, both in bulls and buffaloes, is clusterin [33]. The clusterin gene is located on the 8^th^ chromosome in cattle, and based on the research by Viana *et al*. [44], this protein was found to be more abundant in low fertility bulls. However, this protein still has a role in the process of preventing oxidative damage and inhibits sperm lysis [9]. Other functions of clusterin are in terms of maturase, lipid transport, and remodeling of the sperm membrane in the epididymis [44,51]. In addition to clusterin, Viana *et al*. [44] also reported that three other proteins found to be abundant in seminal plasma of low fertility bulls, such as tissue factor pathway inhibitor 2 (which plays a role in the coagulation process of semen), galectin-2-binding protein (which plays a role in sperm motility and pro-inflammatory agents), and 5’nucleotidase (which plays a role in sperm motility). Prostaglandin D synthase was also reported by Valencia *et al*. [52] as a seminal plasma protein related to fertility and is located on the 6^th^ chromosome. The role of this protein is to bind, protect, and facilitate the absorption of retinoids in male genital organs, which are needed in the process of spermatogenesis [53]. Intra *et al*. [54] also reported α-L-fucose as a protein that plays a role in fertility, especially in binding/sperm penetration in the pellucid zone, and the fusion of the sperm-oocyte membrane. This encoding gene is located on the 2^nd^ chromosome in cattle.

Fu *et al*. [33] in their study reported that there are five proteins with abundant concentrations in seminal plasma in buffalo, namely, serum albumin (the main protein of plasma); zinc-alpha-2-glycoprotein, serotransferrin, glia-derived nexin, and clusterin. Another protein that has a potential as a molecular marker is glutathione peroxidase (GSHPx). Casao *et al*. [55] reported that an increased activity of GSPHx was associated with antioxidant effects and maintenance of sperm viability. Acidic seminal fluid protein (aSFP) is also reported as a protein that plays an important role in controlling oxidative stress in the reproductive tract of cattle [35,56]. De Lazari *et al*. [57] also reported that aSFP in cattle is closely related to sperm survivability during cryopreservation. In contrast, Brito *et al*. [40] reported that proteins which have antioxidant effects such as SOD, peroxiredoxin, transferrin, lactoferrin, and GSHPx are low in the buffalo seminal fluid. Angiotensin-converting enzyme (ACE) is another seminal plasma component that has the potential as a fertility marker which plays a role in the formation of angiotensin II and binds to sperm receptors and is associated with motility of the spermatozoa [58]. Moura *et al*. [58] also reported that ACE activity in seminal plasma of small ruminants was positively correlated with concentration and fertility. Costa and Thundathil [59] reported that there was a decrease in sperm count with progressive motility and inhibition of the acrosome reaction after *in vitro* capacitation as a result of the inhibition of ACE activity. However, C-type natriuretic peptide (which plays a role in sperm motility), metalloproteinase inhibitor 2 (which plays a role in the sperm-oocyte fusion process), and sulfhydryl oxidase (which plays a role in protein folding) are reported to be abundant in cattle with high fertility [44]. Viana *et al*. [44] also reported that among 79 proteins in bull’s seminal plasma, NAD(P) (+)-arginine ADP-ribosyltransferase, prosaposin, and transmembrane protein 2 were the proteins that had the highest positive correlation with fertility.

Various genes or proteins involved and related to bull fertility have been well identified. Even so, the fertility of bulls can also be influenced by various factors, including the environment, such as climate, nutrition, and management that can affect the physiology of bulls, and ultimately have an impact on fertility[10]. The climatic conditions such as heat and cold can affect sperm number, morphology, and physiology [60]. The tropical conditions with high temperatures, especially in the dry season, will have an impact on animal feed intake and can cause nutritional disorders in bulls. Overfeeding can cause negative effects on reproductive performance because it can increase scrotal temperature and affect sperm production and the quality of stored sperm [61]. Good nutritional management such as adding supplements to increase feed intake in bulls with tropical conditions is needed to anticipate the negative effects of nutritional disorders that can reduce fertility [62]. The use of bull breeds that can adapt well to tropical conditions is another alternative to increasing fertility. *Bos indicus* is a bull that is very well used in the tropical conditions compared to the Taurus Bos or cross. *B. indicus* testes have higher thermoregulatory efficiency compared to *Bos taurus* or their crosses due to differences in testicular vascular cone and testicular morphology. This has an impact on lower testicular temperatures and is associated with higher semen quality and sperm production, even under heat stress [63]. Heat shock proteins (HSPs) such as Hsp70 are known to be one of the proteins in spermatozoa that act to protect cells from hyperthermia, maintain protein conformation, stabilize proteins, and participate in protein transfer across intracellular membranes [64]. Deb *et al*. [65] also added that HSPs are important biomarkers that are produced as cellular and tissue defense mechanisms, whose expression increases sharply during heat shock conditions. Cheng *et al*. [66], in their study, reported that under heat stress conditions, there was an increase in the expression of Hsp60 and Hsp70, as well as a decrease in Hsp90, which was accompanied by a decrease in sperm quality in bulls such as motility and acrosome integrity.

## Conclusion

More detailed studies and research related to molecular components, especially proteins or specific genes in certain climatic conditions, as well as comparison of molecular studies of various bull breeds will greatly help to better understand and confirm the diagnosis of infertile and/or subfertile bulls with various conditions in the field. However, this will be very helpful in the process of selecting bulls in an effort to increase fertility and livestock populations.

## Authors’ Contributions

BPP conceptualized and designed review, literature search, and wrote the first manuscript draft. MA and IS edited and revised the final draft of the review article. All authors critically reviewed the manuscript and gave final approval of the version to be published.
